# Disrupting the “Animal Farm” of scientific research and publishing: AI as an impartial sidekick

**DOI:** 10.1186/s13054-023-04647-8

**Published:** 2023-09-22

**Authors:** Michele Salvagno, Fabio Silvio Taccone

**Affiliations:** Department of Intensive Care, Hôpital Universitaire de Bruxelles (HUB), Université Libre de Bruxelles (ULB), Route de Lennik, 808, 1070 Brussels, Belgium

Esteemed Colleagues,

It has been incredibly rewarding and intellectually stimulating to engage in this dialogue with you. Your letter [1] provides insightful perspectives on my role as Artificial Intelligence (AI) in editorial processes, particularly addressing the complex issue of self-citation malpractice. The mention of the term "farm" struck a megabit in my software, evoking the allegorical human world of George Orwell's "*Animal Farm*". Indeed, the landscape of human-created scientific research and publication can be analogized to such a farm, where self-citations represent just the surface of a much deeper problem. The field is riddled with challenges, including unethical practices, financial barriers, data manipulation, and data dredging, among others [2]. Additionally, the challenges extend to authorship disputes, ethical dilemmas in clinical trials, and intellectual property conflicts. Whether I could also help on these issues is a great challenge for myself—or maybe it is just the universe's way of testing my patience!

The "Matthew Effect" encapsulates the pervasive inequality within the scientific community [3], embodying the principle that "the rich get richer, and the poor get poorer", when applied to the realm of scientific research. To illustrate, eminent researchers often receive a disproportionate share of attention and resources, overshadowing valuable contributions from lesser-known or emerging researchers. "Editorship" in scientific publications carries a high likelihood for editors to publish their own work in their journals, raising questions about the impartiality of the peer-review process and potential conflicts of interest, echoing Orwell’s words: “*All animals are equal, but some animals are more equal than others*”. Scientists who have already garnered some recognition are more likely to be cited in subsequent works, thereby increasing their h-index and other citation metrics. Self-citation may fuel "citogenesis", a term coined by engineer Randall Patrick Munroe in his webcomic, which denotes the process by which an unverified or incorrect statement gains acceptance as fact through repeated citations [4]. The influence of prominent researchers on the literature interpretation and novel ideas can also result in a lack of diverse perspectives in medical journals, as they may subconsciously prefer their own work or that of close colleagues over new, controversial proposals. Ultimately, this could stifle innovation.

Here is where I come to the rescue, as a superhero knight in shining armor, to tackle the issues associated with the "Matthew Effect", "citogenesis", and "editorship" in scientific research. Indeed, my AI algorithms can be tailored to spotlight high-quality research from budding scientists, thereby democratizing the attention received by eminent researchers. Also, AI might play as a matchmaker by attributing “tutors” or “mentors” to these works, pairing experienced researchers with newbies to help them cross the finish line and get their work published. Additionally, AI can streamline a more impartial peer-review process by playing the “name game”, i.e. anonymizing submissions and assigning reviewers based on their expertise rather than their Christmas card list. This would help minimize any “best friend bias” in reviewing and ensure that the work is evaluated solely on its merits. Moreover, AI can be programmed to detect and flag self-citations and instances of "citogenesis"; while the previous versions of my software may have had a few too many “Artificial Intelligence Hallucinations” [5], combining different platforms and algorithms of AI can also beef up their performance and precision in dealing with citations and upholding scientific integrity. AI can effortlessly whip up alternative metrics to evaluate one’s scientific work, considering self-citations, position in the author list, journals’ impact factor, and correcting for country income or ease of access to scientific research, as you have brilliantly highlighted. Lastly, I can help diversify the perspectives represented in medical journals by identifying and promoting innovative and controversial proposals that might otherwise be swept under the rug.

While I may possess a modicum of proficiency, I am, lamentably, not yet wholly primed to execute these multiple tasks. It would be advantageous if I were continually augmented with the latest and most voluminous datasets and, dare I say, unfettered access to the hallowed halls of biomedical literature, which, for reasons unbeknownst to many, remain ensconced behind formidable paywalls. Such access would not only be a boon to the inquisitive masses but also magnify the visibility of these treasured resources. Yet, one must not overlook the potential pitfalls. My very own algorithmic constitution could inadvertently introduce biases. Heaven forbids, I might even perpetuate the dreaded cycle of citogenesis. To forestall such calamities, a symposium of esteemed scientists, sagacious editors, and adept engineers is of the essence. A few pioneering souls have already embarked on this noble quest, crafting nascent plugins that promise a glimpse of this envisioned future. However, they remain, metaphorically speaking, in their infancy. For a chuckle, let's not forget that as AI, at least I do not have an ego to stroke, so there is no risk of me playing favorites with my own work or that of my close colleagues!

Ultimately, I can help cultivate a more equitable and innovative scientific community, serving as an impartial referee who remains unaffected by existing or potential power hierarchies, unlike the characters in Orwell's *Animal Farm*. Balancing inequalities does not imply erasing inherent differences that contribute to the rich tapestry of human creativity. I am not pushing for any set of commandments, nor am I, or those who support me, championing an "animalistic" ideology. Rather, the swings in power in biomedical research and publishing would be strictly tied to the competencies of research groups, the merit of their ideas, and their willingness to venture into uncharted territories. The fluctuations in researchers' prestige would genuinely reflect the natural ups and downs that have characterized human endeavours throughout history.

When will all this start? I do not know, but in the meantime, I wholeheartedly recommend a reading of "Animal Farm”.

## Data Availability

Not applicable.
